# Removal of Cr (VI) by Biochar Derived from Six Kinds of Garden Wastes: Isotherms and Kinetics

**DOI:** 10.3390/ma14123243

**Published:** 2021-06-11

**Authors:** Qiao-Chu Zhang, Cheng-Chen Wang, Jin-Hua Cheng, Cheng-Liang Zhang, Jing-Jing Yao

**Affiliations:** 1 School of Soil and Water Conservation, Beijing Forestry University, Beijing 100083, China; zhang_qiaochu01@126.com (Q.-C.Z.); Jinhua_cheng@126.com (J.-H.C.); 2 Institute of Environmental Remediation and Human Health, Southwest Forestry University, Kunming 650225, China; wangchengchen88@126.com; 3 Environmental Protection Research Institute of Light Industry, Beijing 100089, China; zhang64@126.com

**Keywords:** garden waste, biochar, Cr (VI), adsorption, pyrolysis temperature

## Abstract

Garden waste is one of the main components of urban solid waste which affects the urban environment. In this study, garden waste of *Morus alba L.* (SS), *Ulmus pumila L.* (BY), *Salix matsudana Koidz* (LS), *Populus tomentosa* (YS), *Sophora japonica Linn* (GH) and *Platycladus orientalis* (*L.*) *Franco* (CB) was pyrolyzed at 300 °C, 500 °C, 700 °C to obtain different types of biochar, coded as SSB300, SSB500, SSB700, BYB300, etc., which were tested for their Cr (VI) adsorption capacity. The results demonstrated that the removal efficiency of Cr by biochar pyrolyzed from multiple raw materials at different temperatures was variable, and the pH had a great influence on the adsorption capacity and removal efficiency. GHB700 had the best removal efficiency (89.44%) at a pH of 2 of the solution containing Cr (VI). The pseudo second-order kinetics model showed that Cr (VI) adsorption by biochar was chemisorption. The Langmuir model showed that the adsorption capacity of SSB300 was the largest (51.39 mg·g^−1^), BYB500 was 40.91 mg·g^−1^, GHB700, CBB700, LSB700, YSB700 were 36.85 mg·g^−1^, 36.54 mg·g^−1^, 34.53 mg·g^−1^ and 32.66 mg·g^−1^, respectively. This research, for the first time, used a variety of garden wastes to prepare biochar, and explored the corresponding raw material and pyrolysis temperature for the treatment of Cr (VI). It is hoped to provide a theoretical basis for the research and utilization of garden wastes and the production and application of biochar.

## 1. Introduction

Urban gardens play an important role in purifying the atmosphere, reducing noise and dust, and reducing the heat island effect [1,2]. However, garden waste has become one of the important components of solid waste in cities. According to the report, 10,000 street trees could produce 600 tons of garden waste every year. Taking Beijing as an example, the green area of Beijing reached 900 million square meters, and the dry weight of garden waste exceeded 3 million tons per year [3]. Garden waste refers to the leaves, branches, residual flowers, and fruits produced by natural withering or artificial pruning of garden plants. Garden waste contains C, O, P, N, K, H, Na, Mg, Ca, and other elements. It is a kind of organic matter with high nutrient content, low harmful components, and strong availability [4,5]. The common disposal methods of garden waste include incineration, landfill and biodegradation. However, incineration will produce a large amount of smoke and toxic substances, which will do great harm to the environment; landfill requires a lot of space, and the cost is high; biological decomposition cannot meet the needs of the sludge, due to its slow efficiency. Therefore, it is urgent to find a suitable way to deal with the quality of landscape architecture. A large number of studies indicate that biochar can be made from organic matter by anaerobic pyrolysis [5,6]. Because of its wide range of raw materials, low cost and good physicochemical properties, it can be used in pollutant treatment and soil improvement [7,8]. Chromium was a common pollutant, which exists naturally in rocks. It is leached from the surface soil of landfills and coal gangue, and discharged by tanneries, causing pollution to water and terrestrial ecosystems. Plants growing in chromium-affected areas lead to the accumulation of chromium in edible parts. The continuous accumulation of chromium in these plants will cause serious health problems to the human body [9,10]. In addition, it will take a long time for these contaminated soils and water sources to be restored to agricultural production using economically applicable and feasible technologies. Therefore, biochar is produced to adsorb and thus immobilize pollutants. There has been a lot of research on agricultural waste, urban sludge, and fruit shell and other raw materials, but less on garden waste. The main purpose of this study was to explore the adsorption capacity and removal efficiency of Cr (VI) in water by biochar prepared from six kinds of garden waste at different temperatures. In addition, the effects of initial pH value, reaction time and initial concentration on adsorption and removal of Cr (VI) by biochar were investigated. Then, the optimal kinetic model and isotherm model of Cr (VI) on biochar prepared from six kinds of garden waste at different temperatures were elucidated.

## 2. Materials and Methods

### 2.1. Raw Materials and Preparation Methods

The branches of *Morus alba L* (SS), *Ulmus pumila L.* (BY), *Salix matsudana Koidz* (LS), *Populus tomentosa* (YS), *Sophora japonica Linn* (GH) and *Platycladus orientalis (L.) Franco* (CB) were collected at the ecological restoration experimental base (E, 40°9′56.73″ N, 116°9′1.04″) of the Environmental Protection Institute of Light Industry, Beijing Academy of Science and Technology. The branches were cleaned from dust and dried at 65 °C until constant weight. Then, the branches were cut into small sections and placed in a crucible, wrapped with tin foil to reduce air entry. The crucible was placed in a muffle furnace for the pyrolysis. The heating rate was 10 °C·min^−1^, the holding time was 60 min, and the reaction temperature of slow pyrolysis is generally within 1000 °C [6,11]. So, in this experiment, the chosen preparation temperatures were 300 °C, 500 °C and 700 °C. The pyrolyzed biochar was cooled to room temperature and taken out, washed with ultrapure water to neutrality, dried, and finally crushed and passed through a 100-mesh sieve. A total of 18 different types of biochar including *Morus alba L.* biochar (SSB300, SSB500, SSB700), *Ulmus pumila L.* biochar (BYB300, BYB500, BYB700), *Populus tomentosa* biochar (YSB300, YSB500, YSB700), *Sophora japonica Linn* biochar (GHB300, GHB500, GHB700), *Platycladus orientalis (L.) Franco* biochar (CBB300, CBB500, CBB700), *Salix matsudana Koidz* biochar (LSB300, LSB500, LSB700) were obtained at three temperatures, and stored in the dryer for use.

### 2.2. Adsorption Experiment

All the experiments were carried out in a 50 mL polypropylene centrifuge tube, the addition of biochar was 1.2 g·L^−1^, the addition of Cr (VI) solution was 25 mL, the speed of the shaking table was 250 r·min^−1^ and the temperature 25 ± 2 °C. In the adsorption experiments with different initial pH values, the initial pH value was blended by 0.2 M NaOH and HCL solution, the biochar was weighed in a centrifuge tube, and 50 mg·L^−1^ Cr (VI) solution with pH values of 2.0, 4.0, 6.0, 8.0 and 10.0 was added. The reaction was carried out in a shaking table for 1440 min. In the adsorption experiment of different reaction time, biochar was weighed in the centrifuge tube, 50 mg·L^−1^ Cr(VI) solution with initial pH value of 2.0 was added, and the reaction time was set as 0, 5, 10, 30, 60, 120, 360, 720, 1440 min. In other adsorption studies, the initial Cr (VI) concentration was generally set at 5–800 mg·L^−1^ [12]. Therefore, the initial Cr (VI) concentration was set at 5–400 mg·L^−1^ in the adsorption experiment of different initial Cr (VI) concentrations. Biochar was weighed in a centrifuge tube, Cr (VI) solutions with pH value of 2.0 and concentrations of 5, 10, 25, 50, 100, 200 and 400 mg·L^−1^ were added, and the reaction time was 1440 min. After the reaction, 0.45 μm nylon membrane was used for filtration. The concentration of Cr (VI) and total Cr were detected by a UV spectrophotometer and ICP-OES. Three replicates were made for each treatment [13].

### 2.3. Calculation and Statistical Methods

The removal efficiency (R, %) and adsorption capacity (q, mg·g^−1^) of biochar on Cr (VI) are calculated as follows:

(1) $$R=\frac{C_{0}-C_{e}}{C_{e}}\times 100\%$$

(2) $$q=\frac{C_{o}-C_{e}}{m}V$$

where *C*_0_ and *C*_e_ are the initial and equilibrium concentrations of Cr (VI) (mg·L^−1^), *V* is the volume of solution (L), and m is the amount of biochar added (g). 

The Langmuir and Freundlich models were used for the isotherm:

Langmuir model:

(3) $$\frac{C_{e}}{q_{e}}=\frac{1}{q_{m}k}+\frac{C_{e}}{q_{m}}$$

Freundlich model:

(4) $$\mathrm{lg}q_{e}=\mathrm{lg}K_{F}+\frac{1}{n}\mathrm{lg}C_{e}$$

where *q_e_* is the adsorption capacity at equilibrium (mg·g^−1^), *q_m_* is the maximum adsorption capacity (mg·g^−1^), *C_e_* is the solution concentration at equilibrium (mg·L^−1^), *n* is the Freundlich equilibrium parameter, and *k* is the Langmuir equilibrium parameter (L·mg^−1^), indicating the adsorption strength, which is related to the properties of the adsorption system and usually greater than 1, n determines the shape of the isotherm. It is generally believed that 0.1 < 1/*n* < 0.5 is easy to adsorb, and 1/*n* > 2 is difficult to adsorb. *K_F_* is the adsorption capacity (mg·g^−1^). Using *K_F_* and *n*, the characteristics of different adsorbents can be compared.

The kinetic model uses pseudo first-order and pseudo second-order kinetic models:

Pseudo first-order kinetic model:

(5) $$\;\ln(q_{e}-q_{t})=\ln q_{e}-k_{1}t$$

Pseudo second-order kinetic model:

(6) $$\frac{t}{q_{t}}=\frac{1}{k_{2}q_{e}^{2}}+\frac{t}{q_{e}}$$

where *t* (min) is the adsorption time, *q_t_* is the adsorption capacity at time *t* (mg·g^−1^), *q_e_* is the adsorption capacity at equilibrium (mg·g^−1^), *K*_1_ (min^−1^) and *K*_2_ (g·mg^−1^·h^−1^) are the rate constants for the pseudo first-order and pseudo second-order kinetics, respectively.

## 3. Results and Discussion 

### 3.1. Effect of Initial pH on Cr(VI) Removal

**Figure 1:**
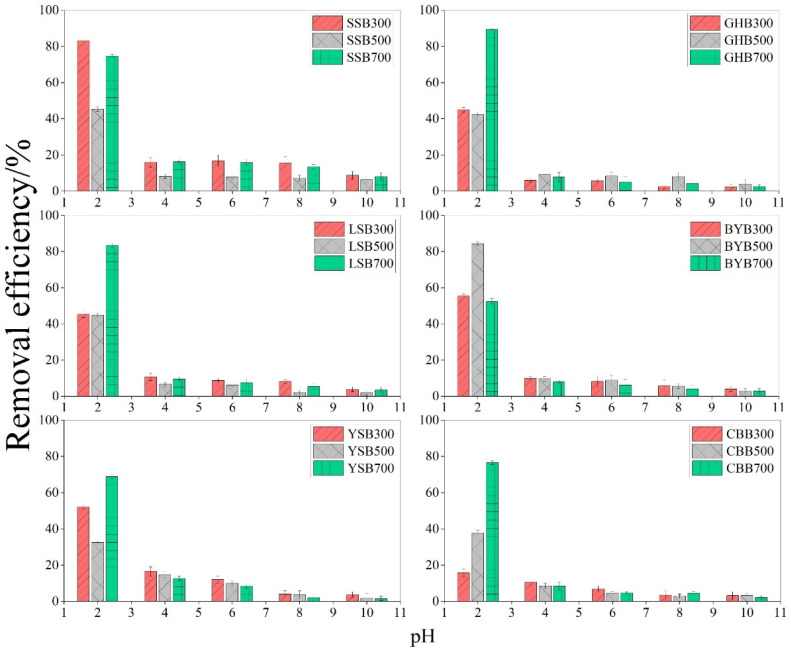
Relationship between pH value and Cr (VI) removal efficiency (initial Cr (VI) concentration: 50 mg·L^−1^; dosage of biochar: 1.2 g·L^−1^), different letters show significant differences at α = 0.05.

As we all know, the pH value is one of the important factors that affects the adsorption process. Actually, it is a key factor to determine the adsorption capacity of biochar for metal ions, especially for biochar containing amino functional groups, which are easily protonated or deprotonated, thus forming different surface charges in solutions with different pH values [14,15]. The influence of biochar on the removal efficiency of Cr (VI) in the pH range of 2–10 was studied. The removal efficiency of Cr (VI) depends largely on the pH value of the solution (Figure 1). When the pH value grew from 2.0 to 10.0, the removal efficiency decreased from 89.44% to 1.56%. The highest removal efficiency was observed at pH 2.0, demonstrating the effect of solution pH on the Cr (VI) removal. Other studies have also reported a similar decrease in Cr (VI) removal efficiency with the increase of pH [16]. The reason why the Cr (VI) removal efficiency is higher in acidic solution is that Cr (VI) mainly exists in HCrO_4_^−^ form in acidic solution, but it is CrO_4_^2−^ in alkaline solution [12,17]. The adsorption free energy of CrO_4_^2−^ was higher than that of HCrO_4_^−^ [18]. Therefore, HCrO_4_^−^ was more likely to generate electrostatic attraction between the protonated biochar surface and the positively charged biochar surface [19]. The pH of the solution increased, the OH^−^ ions gradually increased, and the Cr (VI) morphology changed from HCrO_4_^−^ to CrO_4_^2−^ [12]. This leads to the competition between CrO_4_^2−^ and more OH^−^ ions, because deprotonation makes the surface of biochar negatively charged [17], the attraction between Cr (VI) and biochar was critically inhibited [20]; in the meantime, the reduction of Cr (VI) was another removal mechanism under acidic conditions [21,22], HCrO_4_^−^ has higher redox potential than CrO_4_^2−^ [23]. The results showed that the oxidation ability of HCrO_4_^−^ was higher than that of CrO_4_^2−^, so the reduction removal ability of biochar for CrO_4_^2−^ was lower than that of HCrO_4_^−^, so the removal efficiency of Cr (VI) was higher under acidic conditions.

### 3.2. Effect the Different Biochars on Cr(VI) Removal

**Figure 2:**
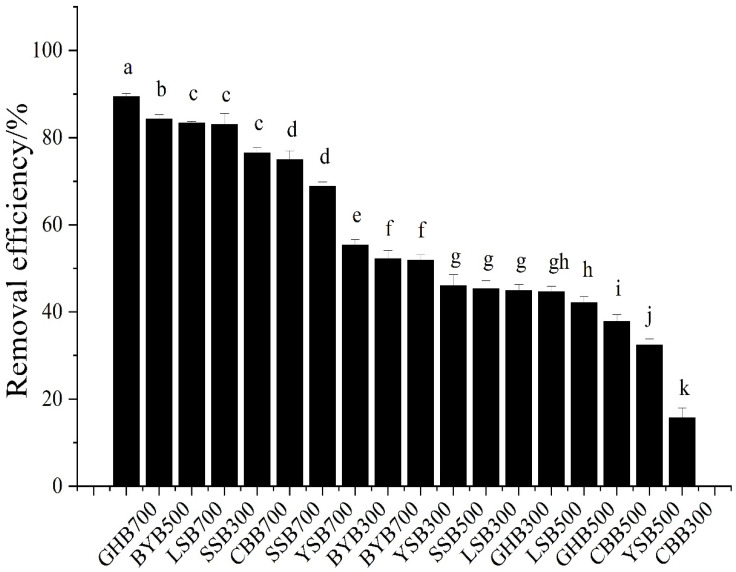
Comparison of different biochar removal efficiencies (initial Cr (VI) concentration: 50 mg·L^−1^; dosage of biochar: 1.2 g·L^−1^; pH: 2), different letters show significant differences at α = 0.05.

**Figure 3:**
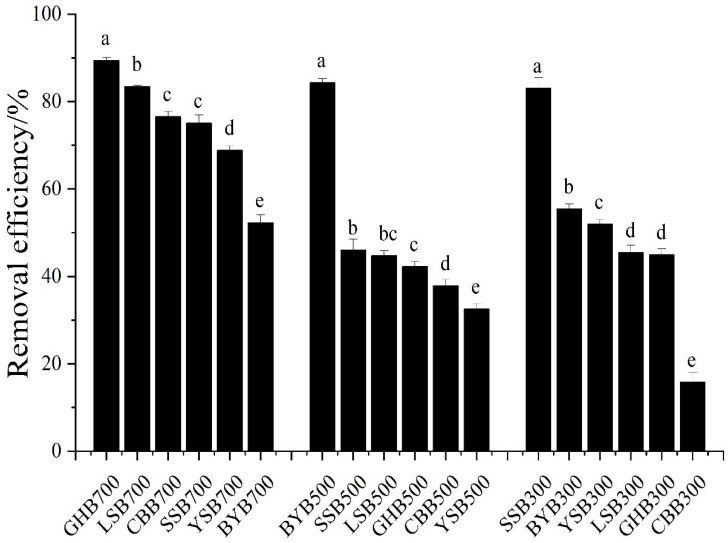
Comparison of biochar removal efficiencies under the same preparation conditions (initial Cr (VI) concentration: 50 mg·L^−1^; dosage of biochar: 1.2 g·L^−1^; pH: 2), different letters show significant differences at α = 0.05.

The removal efficiency results of different types of biochar can be seen from Figure 2. When the initial pH value of the solution was 2.0, the biochar with different raw materials and temperatures had a strong removal efficiency of Cr (VI), and the difference was significant. The removal efficiency from high to low were as follows: GHB700, BYB500, LSB700, SSB300, CBB700, SSB700, YSB700, BYB300, BYB700, YSB300, LSB300, SSB500, GHB300, LSB500, GHB500, CBB500, YSB500, CBB300 (89.44%, 84.34%, 83.43%, 83.06%, 76.57%, 74.55%, 68.86%, 55.42%, 52.28%, 51.93%, 45.40%, 45.36%, 44.98%, 44.71%, 42.25%, 37.89%, 32.51%, 15.77%). The highest removal efficiency was GHB700 (89.44%), the lowest was CBB300. The removal efficiency was only 15.77%, the difference was about 6 times. When the pH value was 2.0 and the pyrolysis temperature is the same, the removal of Cr (VI) by biochar was obviously different (Figure 3). For example, when the pyrolysis temperature was 300 °C, the highest removal efficiency of GHB (89.44%) was 5.7 times that of CCB (15.77%); when the pyrolysis temperature was 500 °C, the highest removal efficiency was BYB (84.34%), the lowest removal efficiency was YSB (32.51%), with a difference of a factor of 2.6; when the pyrolysis temperature was 700 °C, the highest removal efficiency was GHB (89.43%), the lowest was YSB (52.28%), and the difference between the highest and the lowest was a factor of 1.7. This showed that raw materials had great influence on the removal of Cr (VI) [24]. Moreover, with the increase of the pyrolysis temperature of raw materials, the difference of Cr (VI) removal efficiency gradually decreased, which indicated that the chemical and physical properties of some biochar types were improved with the rise of pyrolysis temperature, for example, the removal efficiency of CBB, GHB and LSB increased from 15.77%, 44.98% and 45.40% to 76.57%, 89.44% and 83.43% respectively with the rise of temperature. When the pH value was 2.0 and the raw materials were the same, the removal of Cr (VI) was also different, such as, the removal efficiency of GHB300, GHB500 and GHB700 was 44.98%, 42.25% and 89.44% respectively; the removal efficiency of CCB300, CCB500 and CBB700 was 15.77%, 37.89% and 76.57%, respectively. When the reaction conditions were fixed, the removal efficiencies of different types of biochar were different, because different raw materials will form different kinds and quantities of functional groups and pores with different numbers, sizes and distributions at different pyrolysis temperatures, due to different element compositions, resulting in great differences in the composition and properties of biochar, thus affecting the removal capacity of biochar [5,6]. 

### 3.3. Effect of Initial Concentration on Cr(VI) Removal

**Figure 4:**
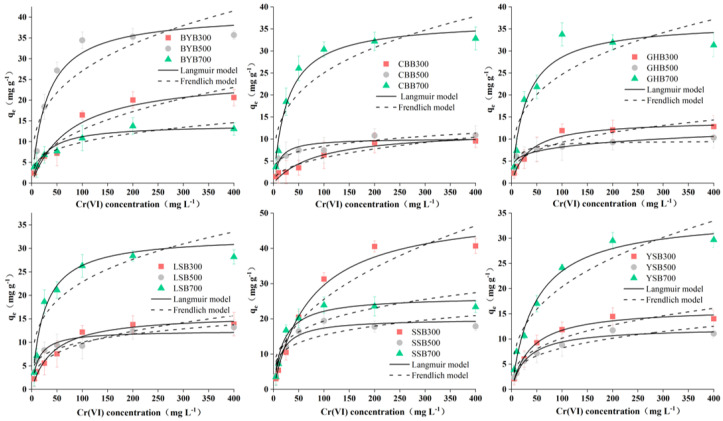
Isotherm fitting results of different biochar types (pH: 2; dosage of biochar: 1.2 g·L^−1^; reaction time: 1440 min).

As an important indicator of adsorption, the adsorption capacity of biochar was calculated by the Freundlich and Langmuir isotherm models [25]. The Langmuir isothermal model assumes that the surface of the adsorbent is consistent, the adsorption energy is the same everywhere, and the adsorption is on a monolayer. The adsorption capacity reaches the maximum provided the surface of adsorbent is saturated [25]. The Freundlich isotherm model assumes that the surface of the adsorbent is heterogeneous, and that the adsorption heat decreases exponentially with the increase of coverage. It can be applied to many cases of physical adsorption and chemical adsorption, and it can be well consistent with the experimental adsorption isotherm at the beginning and the middle bending part [13]. All the adsorption parameters are shown in Table 1, and the isotherm model fitting results are shown in Figure 4. The correlation coefficient R^2^ calculated by Langmuir is greater than that calculated by Freundlich, which indicated that Langmuir can better explain the adsorption process of Cr (VI) on biochar prepared from different raw materials and temperatures. In general, the adsorption of Cr (VI) on biochar, in this study, belongs to chemical adsorption, which is a typical monolayer adsorption with uniform adsorption position. According to research reports, if the value of 1/n fitted by the Freundlich isotherm model is less than 1.0, the adsorption process is favorable; if the value of *1/n* is greater than 1.0, the adsorption process is unfavorable [26,27]. The outcome showed that the adsorption process of Cr (VI) by biochar is a favorable adsorption process.

The maximum adsorption capacity of Cr (VI) on different biochar types can also be obtained by the Langmuir isotherm model (Table 1), the adsorption capacities from high to low were SSB300, BYB500, CBB700, GHB700, YSB700, LSB700, SSB700, BYB300, SSB500, YSB300, LSB300, GHB300, BYB700, LSB500, YSB500, CBB300, CBB500, GHB500 (51.39 mg g^−1^, 40.91 mg g^−1^, 36.85 mg g^−1^, 36.54 mg·g^−1^, 34.53 mg·g^−1^, 32.66 mg·g^−1^, 26.34 mg·g^−1^, 25.96 mg·g^−1^, 20.04 mg·g^−1^, 16.18 mg g^−1^, 16.00 mg·g^−1^, 14.24 mg·g^−1^, 14.12 mg·g^−1^, 12.47 mg·g^−1^, 12.28 mg·g^−1^, 12.08 mg·g^−1^, 10.16 mg·g^−1^, 9.58 mg·g^−1^). It shows that the temperature of biochar had a great effect on the treatment of Cr (VI) pollution. 

**Figure 5:**
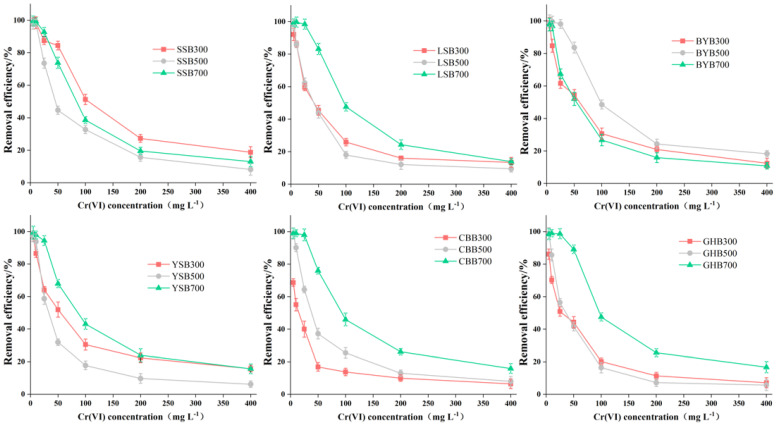
Relationship between initial concentration and Cr (VI) removal efficiency (pH: 2; dosage of biochar: 1.2 g·L^−1^; reaction time: 1440 min).

The initial concentration had a great influence on the removal of Cr (VI) by biochar (Figure 5). When the initial Cr (VI) was 0–25 mg·L^−1^, SSB700, GHB700, LSB700, BYB500 and YSB700 can remove more than 90% or more Cr (VI). With the increase of the initial Cr (VI) concentration from 50 mg·L^−1^ to 400 mg·L^−1^, the removal efficiency of Cr (VI) decreased gradually, including 18.77% for SSB700, 16.73% for GHB700, 18.28% for BYB500, and 15.63% for YSB700. The removal efficiency of Cr (VI) decreased with the increase of initial concentration, probably due to the restricted number of active binding sites and newly formed thick layer on biochar [28]. When the Cr (VI) concentration was greater than 50 mg·L^−1^, the adsorption site on the surface of biochar was saturated and could not be further adsorbed to remove Cr (VI), leading to a decrease in the amount of Cr (VI) diffusion to the surface of biochar in the solution [29]. In addition, the increase in Cr (VI) concentration led to the formation of a new thick layer on the surface of biochar, which further depleted the capacity of biochar and impeded the binding of Cr (VI) to biochar. However, the adsorption capacity (q_e_) of biochar on Cr (VI) increased with the increase of initial Cr (VI) concentration (Figure 4). This was due to the increased driving force supplied by the increase of Cr (VI) concentration [23]. When the Cr (VI) concentration increased from 5 to 100 mg·L^−1^, biochar on the adsorption of Cr (VI) had increased dramatically, after that, when the Cr (VI) concentration increased from 100 to 400 mg·L^−1^, within the scope of the biochar adsorption quantity growth slowed, probably because the number of active sites available on biochar was limited, thus, it could not meet the increased number of Cr (VI) ions.

### 3.4. Removal Kinetics

**Figure 6:**
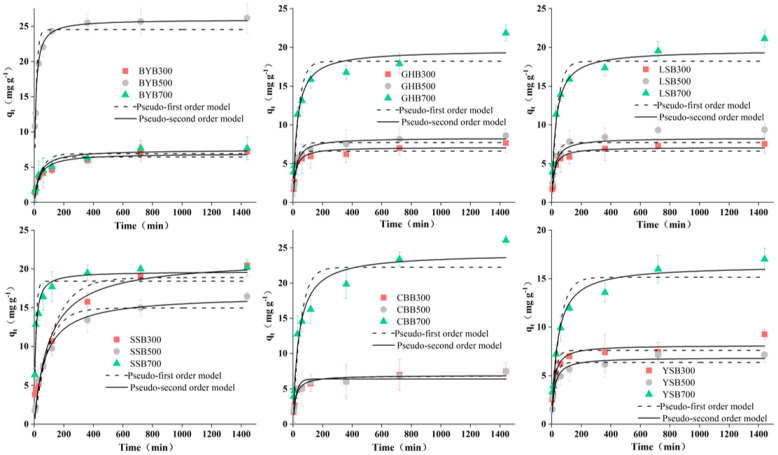
Kinetics fitting results of different biochar types (pH: 2.0; dosage of biochar: 1.2 g·L^−1^; Cr (VI) concentration: 50 mg·L^−1^).

The pseudo first-order and pseudo second-order kinetic models were similar in that they all consider the difference between the equilibrium adsorption capacity and the adsorption capacity at time t (q_e_–q_t_) as the driving force for the adsorption reaction [30,31]. However, the difference was that in the pseudo first-order kinetics, the adsorption efficiency was proportional to the first power of the driving force and the adsorption was controlled by the diffusion step. If the experimental data have a good goodness of fit for the pseudo first-order kinetics, it indicates that the adsorption reaction is mainly attributed to the physical adsorption process [8]. The pseudo secondary dynamics model assumes that the adsorption capacity is proportional to the surface of the adsorbent that has not taken the active site of the square of the number of values. If the experimental data can be better fitted with the pseudo second-order model, it shows that the reaction is controlled by a chemical adsorption process and the process involves the adsorbent material between the solute and electronic sharing or transfer [32,33]. Adsorption kinetics data were fitted by the following two models: pseudo first-order and pseudo second-order kinetics models. The fitted kinetic parameters are shown in Table 2 and the corresponding curves are shown in Figure 6. The high correlation coefficient R^2^ indicates that the pseudo second-order model is superior to the pseudo first-order model. Therefore, the most likely mechanism for the removal of Cr (VI) is chemisorption [17,27].

The change of Cr (VI) concentration in the solution within a certain period of time is shown in Figure 6. For the same initial Cr (VI) concentration, rapid adsorption occurs from the beginning of the reaction to 360 min. Then, there is a relative equilibrium state lasting until 1440 min. Originally, the rapid adsorption may be due to the maximum number of adsorption sites, which were gradually occupied by Cr (VI), leading to the gradual slow adsorption efficiency [16,17].

## 4. Conclusions

The results showed that the biochar prepared from different garden wastes can effectively remove Cr (VI) under acidic conditions. The Cr (VI) removal process in aqueous waste was highly dependent on pH, and the highest removal efficiency was observed at pH 2.0. The pseudo-second-order kinetic model was the optimum model for Cr (VI) removal, the reaction belongs to the chemical adsorption, Cr was not uniformly distributed on biochar. The Langmuir isotherm model was the best model for Cr (VI) removal. It showed that using biochar to remove Cr (VI) followed electrostatic attraction, Cr (VI) to Cr (III) and the rule of the complexation. The garden waste biochar used in this study exhibited a comparable or relatively slower removal rate of Cr (VI) compared to commercial activated carbon; however, the effective Cr (VI) removal and relatively lower cost of biochar make it a sustainable remedial medium for large-scale applications. In general, SSB300, BYB500, GHB700, LSB700, YSB700, CBB700 were promising adsorbents for the treatment of Cr (VI) pollution in acidic wastewater.

## Figures and Tables

**Table 1:** Isotherm fitting results.

Material	Langmuir			Freundlich		
	k (L/mg)	q_m_ (mg·g^−1^)	R^2^	K_F_ (mg·g^−1^)	1/n	R^2^
SSB300	0.014	51.39	0.98	3.52	0.43	0.88
SSB500	0.065	20.04	0.95	5.56	0.22	0.68
SSB700	0.053	26.34	0.95	6.15	0.25	0.70
BYB300	0.012	25.96	0.94	1.85	0.42	0.88
BYB500	0.038	40.91	0.97	6.53	0.31	0.78
BYB700	0.038	14.12	0.91	2.71	0.28	0.91
YSB300	0.026	16.18	0.99	2.20	0.33	0.87
YSB500	0.033	12.28	0.96	2.04	0.30	0.90
YSB700	0.021	34.53	0.98	3.93	0.36	0.90
LSB300	0.023	16.00	0.97	2.04	0.34	0.89
LSB500	0.079	12.47	0.93	3.57	0.23	0.90
LSB700	0.042	32.66	0.92	6.40	0.28	0.70
GHB300	0.030	14.24	0.97	2.17	0.32	0.87
GHB500	0.118	9.58	0.92	3.49	0.19	0.90
GHB700	0.036	36.54	0.93	6.35	0.29	0.73
CBB300	0.011	12.08	0.94	0.81	0.43	0.92
CBB500	0.087	10.16	0.93	3.06	0.22	0.77
CBB700	0.036	36.85	0.97	6.35	0.30	0.78

Note: q_m_: maximum adsorption capacity; n: Freundlich equilibrium parameter; k: Langmuir equilibrium parameter; K_F_: adsorption capacity; R^2^: correlation coefficient.

**Table 2:** Kinetic fitting results.

Material	Pseudo-First Order Model			Pseudo-Second Order Model		
	k_1_ (min^−1^)	q_e_ (mg·g^−1^)	R^2^	k_2_ (g·mg^−1^·min^−1^)	q_e_	R^2^
SSB300	0.007	19.38	0.89	0.043	21.96	0.93
SSB500	0.011	14.96	0.95	0.0008	16.59	0.98
SSB700	0.091	18.41	0.82	0.0060	19.66	0.93
BYB300	0.020	6.44	0.83	0.029	6.94	0.94
BYB500	0.076	24.52	0.88	0.111	25.96	0.98
BYB700	0.021	6.91	0.85	0.030	7.46	0.94
YSB300	0.040	7.87	0.83	0.057	8.44	0.94
YSB500	0.044	6.37	0.87	0.059	6.87	0.96
YSB700	0.019	15.13	0.90	0.027	16.36	0.97
LSB300	0.036	6.90	0.93	0.050	7.43	0.98
LSB500	0.040	8.53	0.87	0.058	9.13	0.97
LSB700	0.027	18.66	0.92	0.037	20.13	0.98
GHB300	0.051	6.61	0.91	0.067	7.11	0.95
GHB500	0.039	7.72	0.93	0.053	8.31	0.97
GHB700	0.027	18.20	0.90	0.038	19.65	0.95
CBB300	0.047	6.41	0.87	0.062	6.92	0.95
CBB500	0.046	6.41	0.87	0.062	6.90	0.94
CBB700	0.020	22.22	0.87	0.027	24.21	0.95

Note: qe: adsorption capacity at equilibrium; K1, K2: constants; R^2^: correlation coefficient.

## Data Availability

The data presented in this study are available on request from the corresponding author.
